# Non-radiologist-performed abdominal point-of-care ultrasonography in paediatrics — a scoping review

**DOI:** 10.1007/s00247-021-04997-x

**Published:** 2021-04-10

**Authors:** Elsa A. van Wassenaer, Joost G. Daams, Marc A. Benninga, Karen Rosendahl, Bart G. P. Koot, Samuel Stafrace, Owen J. Arthurs, Rick R. van Rijn

**Affiliations:** 1 Emma Children’s Hospital, Amsterdam UMC, Paediatric Gastroenterology, University of Amsterdam, Meibergdreef 9, 1105AZ Amsterdam, The Netherlands; 2 Amsterdam Reproduction and Development, Amsterdam UMC, University of Amsterdam, Amsterdam, The Netherlands; 3 Amsterdam Gastroenterology and Metabolism,Amsterdam UMC, University of Amsterdam, Amsterdam, The Netherlands; 4 Amsterdam UMC, Medical Library, University of Amsterdam, Amsterdam, The Netherlands; 5 Department of Radiology, Section of Paediatric Radiology, University Hospital North Norway, Tromsø, Norway; 6 Department of Clinical Medicine, Faculty of Health Sciences, UiT the Arctic University of Norway, Tromsø, Norway; 7 Division of Body imaging, Department of Diagnostic Imaging, Sidra Medicine and Weill Cornell Medicine, Doha, Qatar; 8 Department of Radiology, Great Ormond Street Hospital for Children NHS Foundation Trust, London, UK; 9 NIHR Great Ormond Street Biomedical Research Centre, London, UK; 10 Amsterdam UMC, Radiology, University of Amsterdam, Amsterdam, The Netherlands

**Keywords:** Abdomen, Children, Non-radiologist, Point-of-care, Scoping review, Training, Ultrasound

## Abstract

**Background:**

Historically, US in the paediatric setting has mostly been the domain of radiologists. However, in the last decade, there has been an uptake of non-radiologist point-of-care US.

**Objective:**

To gain an overview of abdominal non-radiologist point-of-care US in paediatrics.

**Materials and methods:**

We conducted a scoping review regarding the uses of abdominal non-radiologist point-of-care US, quality of examinations and training, patient perspective, financial costs and legal consequences following the use of non-radiologist point-of-care US. We conducted an advanced search of the following databases: Medline, Embase and Web of Science Conference Proceedings. We included published original research studies describing abdominal non-radiologist point-of-care US in children. We limited studies to English-language articles from Western countries.

**Results:**

We found a total of 5,092 publications and selected 106 publications for inclusion: 39 studies and 51 case reports or case series on the state-of-art of abdominal non-radiologist point-of-care US, 14 on training of non-radiologists, and 1 each on possible harms following non-radiologist point-of-care US and patient satisfaction. According to included studies, non-radiologist point-of-care US is increasingly used, but no standardised training guidelines exist. We found no studies regarding the financial consequences of non-radiologist point-of-care US.

**Conclusion:**

This scoping review supports the further development of non-radiologist point-of-care US and underlines the need for consensus on who can do which examination after which level of training among US performers. More research is needed on training non-radiologists and on the costs-to-benefits of non-radiologist point-of-care US.

**Supplementary Information:**

The online version contains supplementary material available at 10.1007/s00247-021-04997-x.

## Introduction

In paediatric medicine, US is a widely used imaging technique because it is noninvasive, safe and fast. Traditionally, US examinations are performed by radiologists and ultrasonographers. However, with the introduction of affordable and portable US systems, US is increasingly used as a bedside tool, or the so-called point-of-care, by non-radiologists.

To ensure good medical care for children, a high-quality US examination is of great importance, regardless of the type of physician performing the examination. This quality can be achieved by setting national and international quality standards, and by achieving consensus among US performers on who can do which examination after which level of training. At this point, there is a lack of consensus. This can partly be explained by radiologists, including paediatric radiologists, expressing their fear of losing territory. As the European Society of Radiology (ESR) position paper on US stated, “Turf battles about the use of US continue to grow as more and more specialists are claiming US as part of their everyday’s [sic] work, and the position of radiologists is progressively further undermined” [1]. As a result, non-radiologist point-of-care US has primarily developed outside the sight of radiologists, and consequently many radiologists are not aware of the status of such testing.

If radiologists and non-radiologists would be more aware of both the current uses of non-radiologist point-of-care US and the current gaps in literature, this might form a strong scientific basis for a proper consultation between the two. In a first step to address this issue, we conducted a scoping review focusing on abdominal point-of-care US performed by non-radiologists in children. The aim of this review was to gain an overview of uses of abdominal non-radiologist point-of-care US in children. Additionally, we aimed to identify gaps in the evidence, which can form the basis for future research projects to create a firm scientific base for the implementation of non-radiologist point-of-care US in paediatric medicine.

## Materials and methods

The method for this scoping review was based on the framework outlined by Arksey and O’Malley [2]. The review included the following five key elements: (1) identifying the research question; (2) identifying relevant studies; (3) selecting studies; (4) charting the data; and (5) collating, summarising and reporting the results. The research topics we focussed on were:

- providing an overview of the uses of abdominal non-radiologist point-of-care US, sorted by organ;
- assessing the quality of examinations and training for abdominal non-radiologist point-of-care US;
- assessing the patient perspective of abdominal non-radiologist point-of-care US;
- financial costs of abdominal non-radiologist point-of-care US; and
- legal consequences following the use of abdominal non-radiologist point-of-care US.

The search was conducted with the help of a clinical librarian (J.G.D.) on April 25, 2019, in the Medline, Embase and Web of Science Conference Proceedings databases. The search terms are shown in Online Supplementary Material 1. The inclusion criteria were original research studies on abdominal non-radiologist point-of-care US in children. We excluded studies not written in English, not published, not from Western countries (i.e. North America, Australia or Europe), studies in which both adults and children were studied but in which the data could not be separated, and studies of which no full text was available. In case the US operator was not specified and no radiologist was involved in the study, we assumed the US operator was a non-radiologist. In all other cases, the study was excluded. The full details of the study selection and data extraction can be found in the previously published review protocol [3]. We focussed only on abdominal non-radiologist point-of-care US because given the broadness of the field of non-radiologist point-of-care US, it was not feasible to perform a scoping review of the whole field (e.g., chest or musculoskeletal US).

## Results

**Fig. 1:**
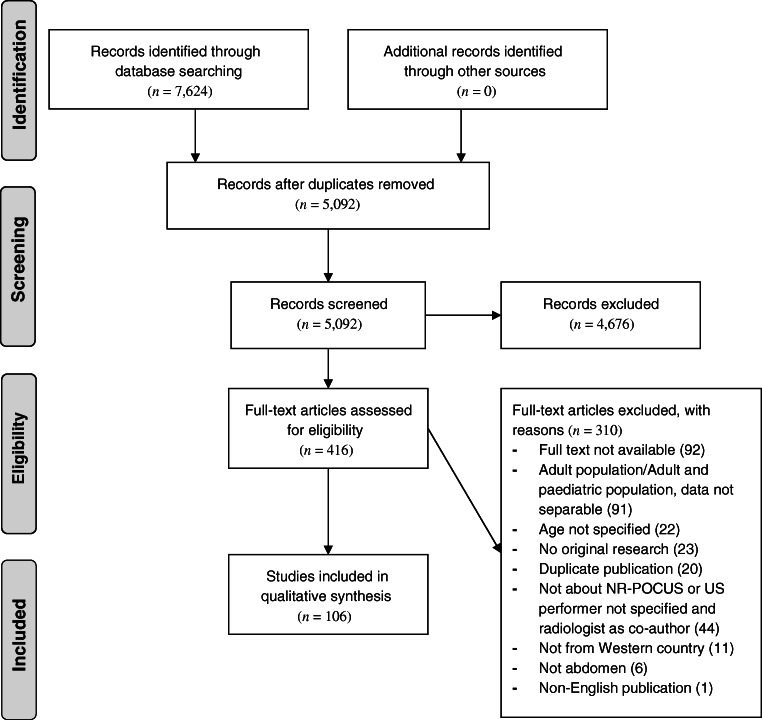
PRISMA (preferred reporting items for systematic reviews and meta-analyses) flowchart. *NR-POCUS* non-radiologist point-of-care ultrasound

The total number of records found from the initial database searches was 7,624. After eliminating 2,532 duplications and subsequently excluding 4,676 records that did not comply with our inclusion criteria based on title and abstract, the number of potentially relevant records was further reduced to 416. Finally, after full-text screening, we included 106 articles: 39 studies and 51 case reports or case series that together gave an overview of the uses of abdominal non-radiologist point-of-care US, 14 on training of non-radiologists, and 1 each on legal consequences following non-radiologist point-of-care US and on patient satisfaction (Fig. 1). No studies on the financial costs of non-radiologist point-of-care US were identified.

**Fig. 2:**
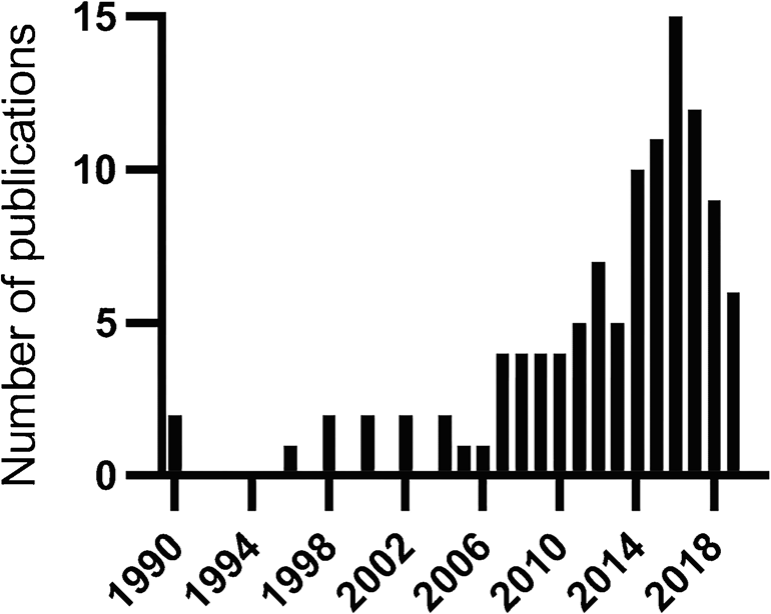
Number of publications on abdominal non-radiologist US in children per year

The 106 articles included in this scoping review were published between 1990 and 2019, with 50 (47%) articles published within the last 5 years (Fig. 2). Most of the studies were conducted in the United States (83%). Only four articles were published in journals with a focus on imaging, two of which were in a journal dedicated to point-of-care US in any environment or setting [4–7]. In 11 articles (10%), a radiologist was named as a co-author.

### Overview of uses of non-radiologist point-of-care ultrasound

Of the 39 studies on abdominal non-radiologist point-of-care US, we found 9 studies on the bladder (Table 1; [8–16]), 10 on the bowel (Table 2; [17–26]), 4 on the stomach (Table 3; [27–30]), 1 on the kidney (Table 4; [31]), 4 on fluid status (Table 5; [4, 32–34]), 9 on non-radiologist point-of-care US for trauma screening (Table 6; [35–43]) and 1 “other” on umbilical artery line placement (Table 7; [44]). Next we present these studies per organ. The case reports and series are displayed in Table 8 [5, 45–94].

**Table 1:** Characteristics of included studies on non-radiologist US of the bladder

Author	Year	Country	Department	*n*	Age (years)	Design	Indication	Ultrasound performer^a^
Massagli [8]	1990	USA	Paediatrics	20	0–16	Obs	Bladder volume	–
Chen [9]	2005	USA	Emergency	136	0–2	Obs	Bladder volume	Paediatric EDP
Witt [10]	2005	USA	Emergency	65	0–1	RCT	Bladder volume	–
Baumann [11]	2008	USA	Emergency	45	0–1	RCT	Bladder volume	Nurse
Dessie [12]	2018	USA	Emergency	120	14	RCT	Bladder volume	Paediatric EDP
Weill [13]	2019	Canada	Emergency	201	0	RCT	Bladder volume	Paediatric EDP
Enright [14]	2010	UK	Emergency	45	1–4	Obs	Dehydration	–
Buntsma [15]	2012	Australia	Emergency	60	0–1	Obs	Suprapubic aspiration	EDP
Gochman [16]	1991	USA	Emergency	66	0–1	RCT	Suprapubic aspiration	EDP

*EDP* emergency department physician, *Obs* observational, *RCT* randomised controlled trial

– indicates not reported, and no radiologist as co-author

**Table 2:** Characteristics of included studies on non-radiologist US of the bowel

Author	Year	Country	Department	*n*	Age (years)	Design	Indication	Ultrasound performer
Fox [17]	2008	USA	Emergency	48	7–18	Obs	Appendicitis	EDP
Elikashvili [18]	2014	USA	Emergency	150	12 (SD 5.2)	Obs	Appendicitis	Paediatric EDP
Doniger [19]	2018	USA	Emergency	40	2–18	Obs	Appendicitis	EDP
Nicole [20]	2018	Canada	Emergency	121	8–14	Obs	Appendicitis	EDP
Soundappan [21]	2018	Australia	Surgery	65	3–15	Obs	Appendicitis	Paediatric surgeon
Jimbo [22]	2016	Japan	Surgery	84	4–15	Retro	Appendicitis	Paediatrician
Riera [23]	2012	USA	Emergency	82	0–10	Obs	Intussusception	EDP
Lam [24]	2014	USA	Emergency	44	2	Retro	Intussusception	EDP
Gurien [25]	2017	USA	NICU	17	0	Obs	Motility	Surgeon
Doniger [26]	2018	USA	Emergency	50	4–17	Obs	Constipation	Clinicians

*EDP* emergency department physician, *NICU* neonatal intensive care unit, *Obs* observational, *Retro* retrospective, *SD* standard deviation

**Table 3:** Characteristics of included studies on non-radiologist US of the stomach

Author	Year	Country	Department	*n*	Age (years)^a^	Design	Indication	Ultrasound performer
Schmitz [27]	2016	Switzerland	Anaesthesiology	18	6–12	Obs	Empty stomach	Anaesthesiologist
Moser [28]	2017	Canada	Anaesthesiology	100	8–16	Obs	Empty stomach	Anaesthesiologist
Sivitz [29]	2013	USA	Emergency	67	0 (IQR 14–83 days)	Obs	Pyloric hypertrophy	Paediatric EDP
Wyrick [30]	2014	USA	Surgery	17	–	Obs	Pyloric hypertrophy	Surgeon, paediatric EDP

*EDP* emergency department physician, *IQR* interquartile range, *Obs* observational

– indicates not reported, and no radiologist as co–author

**Table 4:** Characteristics of included studies on non-radiologist US of the kidney

Author	Year	Country	Department	*n*	Age (years)	Design	Indication	Ultrasound performer
Guedj [31]	2015	France	Emergency	433	0 (IQR 0–1)	Obs	Hydronephrosis	EDP

*EDP* emergency department physician, *IQR* interquartile range, *Obs* observational

**Table 5:** Characteristics of included studies on non-radiologist US of fluid status

Author	Year	Country	Department	*n*	Age (years)	Design	Indication	Ultrasound performer
Chen [32]	2007	USA	Emergency	72	0–16	Obs	Dehydration	Paediatric EDP
Chen [33]	2010	USA	Emergency	112	5 (SD 4)	Obs	Dehydration	Paediatric EDP
Jauregui [4]	2014	USA	Emergency	113	0–18	Obs	Dehydration	EDP
Wyrick [34]	2015	USA	Surgery	31	0–0	Obs	Dehydration	Surgeon

*EDP* emergency department physician, *Obs* observational, *SD* standard deviation

**Table 6:** Characteristics of included studies on non-radiologist US after trauma

Author	Year	Country	Department	*n*	Age (years)	Design	Indication	Ultrasound performer^a^
Ingeman [35]	1996	USA	Emergency	31	2–18	Obs	Free fluid	EDP
Thourani [36]	1998	USA	Emergency	192	0–14	Obs	Free fluid	Surgeon
Partrick [37]	1998	USA	Surgery	230	0–17	Obs	Free fluid	Surgeon
Corbett [38]	2000	USA	Emergency	47	9	Obs	Free fluid	EDP
Scaife [39]	2013	USA	Emergency	88	2–12	Obs	Free fluid	Surgeon
Menaker [40]	2014	USA	Emergency	887	6–16	Obs	Free fluid	EDP
McGaha [41]	2019	USA	Emergency	292	11+−5	Retro	Free fluid	–
Holmes [42]	2017	USA	Emergency	925	9.7±5.3	RCT	Free fluid	EDP
Brenkert [43]	2017	USA	Emergency	103	6–14	Retro	Free fluid	EDP

*EDP* emergency department physician, *Obs* observational, *Retro* retrospective, *RCT* randomised controlled trial

– indicates not reported, and no radiologist as co-author

### Bladder

Of the nine studies on non-radiologist point-of-care US of the bladder, six assessed bladder volume, two during suprapubic aspiration; one assessed the degree of dehydration (Table 1). Of the studies regarding bladder volume, we identified four randomised controlled trials and two observational studies, mostly aiming to assess the benefit of using non-radiologist point-of-care US to obtain a valid urine sample for urinalysis. Three studies used success rates of catheterisation in infants as the end point and all found an increased success rate when using non-radiologist point-of-care US prior to catheterization [9–11]. One study used success rate of obtaining a clean-catch urine sample and did not find a difference between the two groups [13], and one study found that performing an non-radiologist point-of-care US prior to sending a child to the radiology department for a transabdominal pelvic US predicted the patient readiness for the examination and decreased time to pelvic US. The two studies regarding suprapubic aspiration both assessed whether the success rate could be improved. One study was a randomised controlled trial comparing blind suprapubic aspiration to non-radiologist point-of-care US-guided suprapubic aspiration and found a higher success rate in the US-guided group (79% vs. 52%, *P*=0.04) [16]. The other study demonstrated a success rate of only 53% when using non-radiologist point-of-care US for bladder scan [15]. Last, the results of the last study suggest that non-radiologist point-of-care US for bladder scan could be used to monitor urine production in children suspected of having dehydration [14].

### Bowel

We identified six studies on non-radiologist point-of-care US for diagnosing appendicitis, two on intussusception, one on constipation and one on bowel motility (Table 2). Six studies assessed the diagnostic accuracy of non-radiologist point-of-care US in diagnosing appendicitis in children, all with a combination of pathology and clinical follow-up details as reference standard [17–21, 26]. For detailed analysis of diagnostic accuracy, we refer to a previously published systematic review on this topic [95]. In two of the included studies, performance of non-radiologists was compared to that of radiologists. One of these two studies demonstrated a comparable accuracy between the two raters and a sensitivity of 82% (95% confidence interval [CI]: 64–92) vs. 96% (95% CI: 83–99) and specificity of 97% (95% CI: 85–99) vs. 100% (95% CI: 87–100), respectively [21]. In contrast, the other study demonstrated that non-radiologists reported inconclusive results more often than radiologists (59% compared to 15%, respectively) [20]. Last, one study showed that the use of non-radiologist point-of-care US could decrease the length of hospital stay for children suspected of having appendicitis (length of stay decreased from 288 min (95% CI: 256–319) to 154 min (95% CI: 113–195) [18].

The two studies regarding intussusception assessed the diagnostic accuracy of non-radiologist point-of-care US, using radiology department examinations as a reference standard (either radiology US [23] or any (i.e. CT, US, barium enema) [24]. Sensitivity of non-radiologist point-of-care US ranged from 85% to 100% and specificity from 97% to 100%.

Finally, one pilot study showed that non-radiologist point-of-care US can be used to detect return of bowel function in infants with gastroschisis by assessing presence of motility [25], and one study assessed whether measuring the transrectal diameter can be used to diagnose constipation in children with abdominal pain. The latter study showed a sensitivity of 86% (95% CI: 69–96), and a specificity of 71% (95% CI: 53–85) using the Rome III criteria as a reference standard [26].

### Stomach

We identified two studies on preoperative gastric content assessment and two on pyloric hypertrophy diagnosis (Table 3). The two studies on non-radiologist point-of-care US regarding the assessment of stomach filling status were from the anaesthesiology department and assessed whether non-radiologist point-of-care US could be used to assess whether a patient can be intubated safely. One of these studies used MRI findings as a reference standard [27] and the other used gastroscopy [28]. Both studies demonstrated that gastric content could be assessed with acceptable accuracy (area under the curve for measurements in the right lateral decubital position ranged from 0.73 to 0.92).

The other two studies demonstrated that non-radiologist point-of-care US is capable of accurately diagnosing pyloric hypertrophy when using radiology US as reference standard (sensitivity when identifying pylorus: 100% [95% CI: 66–100]; specificity, 100% [95% CI: 92–100]) [29]. There was no difference between measurements obtained by the non-radiologists compared to the radiologists (*P*>0.2) [29, 30].

### Kidney

The one study on kidneys assessed the diagnostic accuracy of non-radiologist point-of-care US in diagnosing hydronephrosis. It found a sensitivity of 77% (95% CI: 58–95%) and a specificity of 97% (95% CI: 95–99%), using radiology US as reference standard (Table 4) [31].

### Fluid status

We identified four studies that assessed the use of non-radiologist point-of-care US in determining fluid status (Table 5). All used the inferior vena cava/aorta ratio and compared this ratio to dehydration. Dehydration was assessed by weight loss, clinical judgement of dehydration, or bicarbonate level. Reported sensitivity ranged from 39% to 86% and reported specificity ranged from 56% to 100% [4, 32–34].

### Trauma screening

We identified nine studies on non-radiologist point-of-care US after trauma (i.e. non-radiologist focused abdominal sonography for trauma [FAST]) (Table 6). Four of these studies assessed the diagnostic utility of non-radiologist point-of-care US after trauma using CT, findings during laparoscopy, or clinical outcome as a reference standard. The reported sensitivity ranged 50–100% (95% CI: 36–100) and the specificity ranged 96–100% (95% CI: 80–100) [35, 36, 38, 39]. Five of the identified studies assessed the clinical impact of non-radiologist point-of-care US on management after trauma, either by assessing the impact on number of needed CT scans [37, 39, 40, 42] or by assessing the success rate of nonoperative management (i.e. not needing an intervention) based on the non-radiologist point-of-care US result [41]. Most of these studies demonstrated that, overall, the use of CT decreased when non-radiologist FAST was increasingly used [37, 39, 40]. However, in hemodynamically stable patients, the clinical care (e.g., length of hospital stay and CT usage) did not improve by using non-radiologist FAST [42]. In addition, one study reported that in 5/88 (6%) patients, the non-radiologist FAST exam was negative, whereas the patients had a significant injury (e.g., required blood transfusion) and that in one of these cases the surgeon would have cancelled the CT based on the non-radiologist FAST exam [39].

### Other

We identified one study on a procedural non-radiologist point-of-care US, regarding umbilical artery catheter placement. This study showed that non-radiologist point-of-care US can reduce the time of line placement from 139 min (standard deviation [SD]: 49 min) to 75 min (SD: 25 min) (*P*<0.001) [44] (Table 7).

**Table 7:** Characteristics of included studies on other non-radiologist US for “other” category

Author	Year	Country	Department	*n*	Age (years)	Design	Indication	Ultrasound performer
Fleming [44]	2011	USA	NICU	31	0	RCT	Line placement in umbilical artery	Neonatologist and fellows

*NICU* neonatal intensive care unit, *RCT* randomised controlled trial

### Case reports and case series

We identified 49 case reports and case series on abdominal non-radiologist point-of-care US in children (Table 8). According to these publications, a total of 31 different diagnoses were made with the help of non-radiologist point-of-care US. In all but three publications, the diagnosis was made at the emergency department.

**Table 8:** Case reports and series on abdominal non-radiologist US in paediatrics

Author	Year	Country	Department	*n*	Age (years)	Organ	Diagnosis	Ultrasound performer^a^
Hinds [45]	2015	USA	Emergency	1	4	Abdomen	Lymphangioma	EDP
Dingman [46]	2007	USA	Emergency	1	4	Aorta	Aortic coarctation	Emergency medicine resident
Baumann [47]	2008	USA	Emergency	1	0	Bladder	Bladder volume	Nurse
Elsamra [48]	2011	USA	Urology	2	14–17	Bladder	Ovarian cyst	–
Ng [49]	2015	USA	Emergency	1	3	Bladder	Urolithiasis	EDP
Chandra [50]	2015	USA	Emergency	8	5–17	Bladder	Urolithiasis	Paediatric EDP
Stone [51]	2010	USA	Emergency	1	6	Bowel	Appendicitis	EDP
Halm [52]	2010	USA	Emergency	1	15	Bowel	Appendicitis	Paediatric EDP
Lavine [53]	2014	USA	Emergency	1	8	Bowel	Appendicitis	EDP
Ravichandran [54]	2016	USA	Emergency	1	3	Bowel	Appendicitis	EDP
Pade [55]	2018	USA	Emergency	1	5	Bowel	Appendicitis	Paediatric emergency medicine fellow
Horowitz [56]	2016	USA	Emergency	3	2–4	Bowel	Foreign body	EDP
Leibovich [57]	2015	USA	Emergency	2	2–13	Bowel	Foreign body	–
Ramgopal [58]	2017	USA	Emergency	1	0	Bowel	Free fluid	Paediatric EDP
Alfonzo [59]	2017	USA	Emergency	1	0	Bowel	Hernia	–
Kairam [60]	2009	USA	Emergency	1	0	Bowel	Intussusception	EDP
Alletag [61]	2011	USA	Emergency	1	0	Bowel	Intussusception	Paediatric emergency medicine fellow
Halm [62]	2013	UK	Emergency	1	2	Bowel	Intussusception	EDP
Ramsey [63]	2014	USA	Emergency	1	4	Bowel	Intussusception	Paediatric emergency medicine fellow
Nelson [5]	2014	USA	Emergency	1	6	Bowel	Intussusception	EDP
Doniger [64]	2016	USA	Emergency	2	0–2	Bowel	Intussusception	Paediatric EDP
Sharma [65]	2019	Canada	Emergency	2	0	Bowel	Intussusception	Paediatric emergency medicine fellow
Garcia [66]	2019	USA	Emergency	5	0–9	Bowel	Malrotation/volvulus	Paediatric EDP
Kornblith [67]	2016	USA	Emergency	2	3–15	Bowel	Meckel diverticulitis	EDP
Brazg [68]	2016	USA	Emergency	1	5	Bowel	Omental torsion	EDP
James [69]	2016	Canada	Emergency	5	5–14	Bowel	Small-bowel obstruction	EDP
Sivitz [70]	2013	USA	Emergency	1	0	Bowel	Volvulus	Paediatric EDP
Tsung [71]	2010	USA	Emergency	13	1–15	Gallbladder	Cholecystitis	–
Shihabuddin [72]	2013	USA	Emergency	1	10	Gallbladder	Cholecystitis	Paediatric EDP
Damman [73]	2016	USA	Emergency	1	0	Gallbladder	Cholelithiasis	–
Gilmore [74]	2004	USA	Emergency	1	0	Kidney	Hydronephrosis	EDP
Hall [75]	2011	UK	Emergency	1	13	Kidney	Hydronephrosis	EDP
Schecter [76]	2012	USA	Emergency	1	7	Kidney	Hydronephrosis	–
Dunlop [77]	2014	USA	Emergency	1	9	Kidney	Renal carcinoma	–
Garcia [78]	2019	USA	Emergency	1	7	Kidney	Stent displacement	Paediatric EDP
Ginger [79]	2009	USA	Urology	8	0–17	Kidney	Stent placement	Urologists
Jamjoom [80]	2015	Canada	Emergency	4	0–11	Liver	Neuroblastoma	EDP
Pe [81]	2016	USA	Emergency	1	13	Ovary	cystic adenoma	EDP
Johnson [82]	2006	USA	Emergency	1	15	Ovary	Ovarian torsion	EDP
Pershad [83]	2002	USA	Emergency	1	10	Spleen	Splenic rupture	EDP
Parekh [84]	2018	USA	Anaesthesiology	3	0–3	Stomach	Empty stomach	Anaesthesiologist
Myatt [85]	2018	USA	Emergency	3	2–12	Stomach	Gastric tube placement	EDP
Malcolm [86]	2009	USA	Emergency	8	0	Stomach	Pylorus hypertrophy	EDP
Pershad [87]	2000	USA	Emergency	1	16	Trauma	Free fluid	EDP
Gallagher [88]	2012	USA	Emergency	1	3	Trauma	Free fluid	EDP
Root [89]	2018	USA	Emergency	1	17	Trauma	Free fluid	Paediatric EDP
Godambe [90]	2007	USA	Emergency	1	8	Trauma	Hydronephrosis	EDP
Neville [91]	2017	USA	Emergency	1	16	Trauma	Splenic rupture and liver laceration	Paediatric emergency medicine fellow
Fischer [92]	2014	Canada	Emergency	1	12	Uterus	Haematocolpometra	Paediatric EDP
Gross [93]	2017	USA	Emergency	1	11	Vagina	Foreign body	EDP
Lahham [94]	2016	USA	Emergency	1	16	Vena cava	Thrombus	EDP

*EDP* emergency department physician

– indicates not reported, and no radiologist as co-author

### Quality and training

We identified 16 published articles concerning the training of non-radiologists performing non-radiologist point-of-care US in children (Table 9; [6, 7, 23, 30, 31, 38, 39, 96–104]). We subdivided these publications into three categories: (1) studies reporting efforts and outcomes of general training strategies for non-radiologist point-of-care US, (2) studies reporting training strategies for a dedicated application of non-radiologist point-of-care US and (3) surveys that reported the state of non-radiologist point-of-care US use and training in paediatric medicine. We describe these findings in the following subsections.

**Table 9:** Training strategies (*n*=14)

Author	Year	Country	Department	Design	Training method
Cohen [96]	2012	USA	Emergency	Survey	76% received bedside/informal teaching, 23% received training by lectures and 16% by workshops or full-day course
Conlon [97]	2015	USA	PICU	Prospective training study (general training)	- 2-day introductory course: didactic and hands-on training sessions with max 5 students per trainer; 4 consensus-derived training modules (procedural, hemodynamic, thoracic and abdominal) - Demonstration of skills after >25 acceptable studies per module - Reviewing of POCUS images twice a week by non-radiologist POCUS experts and once a month by radiology department
Corbett [38]	2000	USA	Emergency	Prospective training study (post trauma)	1-day training course: didactic lectures, a videotaped session with instruction on trauma US, videotape with real-time images of pathology, hands-on workshop on healthy volunteers and finally a test using images
Gold [98]	2017	USA	Emergency	Survey	Didactics (70%), simulations in skills lab (52%), structured rotations by trained faculty (39%) or no US education (12%)
Guedj [31]	2015	France	Emergency	Prospective training study (single-organ POCUS)	- 1–2-h didactic session (basics, physics, UTI sonography) - Hands-on training: 5 procedures
Hoeffe [99]	2016	Canada	Emergency	Survey	Radiology rotation (28%), official course (45%), no training (28%)
Kornblith [100]	2015	USA	Emergency	Survey	Not specified
Kwan [101]	2019	Canada	Emergency	Prospective training study (general training)	Via an online POCUS image interpretation learning and assessment system with 100 cases per application (e.g., FAST, lung, cardiac) with acceptable quality and showing a spectrum of pathology and normal anatomy. Included short clinical presentation, a video and image. Trainees could respond if case was normal/abnormal, and in case of abnormal the area of abnormality was to be selected, and they received feedback
Marin [6]	2012	USA	Emergency	Survey	Bedside (40%), general emergency management physician training (40%), formal course (25%), outside CME course (10%), radiology training (8%)
Nguyen [102]	2016	USA	NICU/PICU	Survey	Bedside (63%), lectures (54%), workshops (47/65%), self-study (47/43%), radiology rotation (26/5%) (NPM/PCCM, respectively)
Ramirez–Schremp [103]	2008	USA	Emergency	Survey	US rotation (33%), hands-on experience (33%), conferences (41%)
Reaume [7]	2019	USA	Emergency	Survey	Procedure-only training (34%), rotations in other departments (22%), no US training (12%)
Riera [23]	2012	USA	Emergency	Prospective training study (single-organ POCUS)	- All trainees had >1 month of clinical instruction in performing a variety of POCUS procedures in emergency department (100–150 procedures on adults). No previous experience with bowel US - 1 h focused training session: didactic component and hands-on scanning with child as a model
Scaife [39]	2013	USA	Emergency	Prospective training study (FAST)	- Technical instruction, viewing an instructional video, didactic session including hands-on training - At least 30 exams, of which 5 were proctored by certified paediatric sonographer or certified adult emergency medicine physician and of which at least 5 were positive for abdominal free fluid - Final competence exam (patients with ascites or ventriculoperitoneal shunt). Topics for exam: detection of intra-abdominal fluid, orientation and accuracy of probe placement, adequate scanning through fields, acceptable efficiency/time frame and ability to obtain key structures
Shefrin [104]	2019	USA	Emergency	Delphi procedure	Not applicable
Wyrick [30]	2014	USA	Surgery	Prospective training study (single-organ POCUS)	Five hands-on exams

*CME* continuing medical education, *FAST* focused abdominal sonography for trauma, *NICU* neonatal intensive care unit, *NPM* neonatal perinatal medicine, *PCCM* pediatric critical care medicine, *PICU* paediatric intensive care unit, *POCUS* point-of-care ultrasound, *UTI* urinary tract infection

#### Studies reporting efforts and outcomes of general training strategies for non-radiologist point-of-care ultrasound

The first is a study from a paediatric critical care department that reported initial efforts, structure, and progress within the division and institution to train and credential physicians [97]. Physicians were trained as follows: they first participated in a 2-day introductory course with didactic lectures and hands-on training sessions. The training consisted of four modules: procedural, haemodynamic, thoracic and abdominal. After the training they were encouraged to perform at least 25 point-of-care US exams per module. Images were saved and were reviewed by point-of-care US experts once a week and by a radiologist once a month. Although only one of the 25 trainees completed the whole course, the non-radiologist point-of-care US examinations they performed contributed to the clinical management (i.e. after performing the US the clinical management was changed) and the authors reported a good experience with the reviewing process.

Another study designed an online learning platform to train paediatric emergency medicine physicians and reported the performance of the trainees [101]. The learning platform consisted of 100 cases (including short clinical presentation, video, images) per application (e.g., FAST, lung, cardiac) and trainees had to distinguish pathology from normal anatomy. In case of pathology they had to identify the location. After every case they received feedback. On average participants needed to complete 1–45 cases to reach 80% accuracy and 11–290 cases to reach 95% accuracy. The least efficient participants (95th percentile) needed to complete 60–288 cases to reach 80% accuracy and 243–1,040 to reach 95% accuracy. Most participants needed about 2–3 h to achieve the highest performance benchmark.

The last study in this category was a publication describing the efforts of a number of experts in the field of paediatric emergency medicine non-radiologist point-of-care US to reach consensus on the core applications to include in point-of-care US training for paediatric emergency medicine physicians using the Delphi method [104]. They concluded that applications of abdominal non-radiologist point-of-care US to include in training of non-radiologists were free peritoneal fluid, abscess incision and drainage, central line placement, intussusception, intrauterine pregnancy, bladder volume, and detection of foreign bodies. According to the experts, applications to exclude from training were abdominal aortic aneurism and ovarian torsion.

#### Studies reporting training strategies for a dedicated application of non-radiologist point-of-care ultrasound

Five articles described a training strategy for a dedicated application of non-radiologist point-of-care US. These included teaching paediatric emergency medicine fellows to measure the pyloric channel when hypertrophic pyloric stenosis is suspected [30], teaching emergency physicians to diagnose hydronephrosis in children with a urinary tract infection [31], teaching emergency physicians to diagnose ileocolic intussusception [23], training paediatric trauma surgeons to perform a FAST [39] and teaching emergency physicians to diagnose free abdominal fluid after trauma [38].

For the single-organ non-radiologist point-of-care US examinations (pyloric channel measurement, detecting hydronephrosis and detecting ileocolic intussusception), the training consisted of a short hands-on training (e.g., about five non-radiologist point-of-care US exams) with or without a preceding didactic lecture about US physics and the specific pathology. In these studies trainees were able to detect the specific pathology with acceptable accuracy (sensitivity: 77% [95% CI: 58–95], specificity: 97% (95% CI: 95–99]) at the end of the training [23, 30, 31].

For the multiple-organ non-radiologist point-of-care US examinations (i.e. post-trauma non-radiologist point-of-care US) the training was more extensive. For the detection of free fluid, trainees followed a 1-day training that consisted of didactic lectures, a videotaped session with instruction, real-time images of pathology and a hands-on workshop on healthy volunteers. After the training, trainees were able to detect free fluid in trauma patients with a sensitivity of 75% (95% CI: 36–95) and a specificity of 97% (95% CI: 81–100) [38]. For the FAST training, paediatric surgeons were trained for about 16 months: they first followed a technical instruction and hands-on training and then they had to perform at least 30 FAST exams. After this training they had to complete an exam on patients with known ascites. Sensitivity for significant amounts of free fluid was 50%, and specificity was 85%. In addition, surgeons reported they never felt they became experts, and they judged 4–10% of non-radiologist point-of-care US exams as inconclusive [39].

#### Surveys that reported the state of non-radiologist point-of-care ultrasound use and training in paediatric medicine

We identified eight survey studies, published between 2008 and 2018. All aimed to evaluate current state of non-radiologist point-of-care US use and education in a paediatric department (either paediatric emergency medicine, paediatric critical care medicine or neonatal medicine), all studies were performed in North America [6, 7, 96, 98–100, 102, 103]. From these surveys it becomes clear that the number of paediatric emergency departments using non-radiologist point-of-care US has increased over the last 12 years (from about 57% to 95%). However, all surveys reported a broad variety of training curricula. Reported methods of training were: bedside training, general emergency department training by a non-radiologist point-of-care US experts, following a formal course, a radiology rotation or training in a skills lab. Reported perceived barriers to implement point-of-care US training were mostly lack of training personnel, lack of time, lack of training guidelines, concerns about liability, and resistance from the radiology department.

### Patient perspectives

We identified one study that evaluated the satisfaction with emergency department visits of caregivers of children who received a non-radiologist point-of-care US examination (either for diagnostic or educational purposes) compared to that of children who did not receive a non-radiologist point-of-care US examination (Table 10) [105]. Caregivers’ satisfaction was measured with a visual analogue scale. In this study, there was no difference in satisfaction between patients who did and did not receive a non-radiologist point-of-care US examination, and two-thirds of caregivers reported that they felt the examination improved the child’s interaction with the emergency department physician.

**Table 10:** Patient perspectives

Author	Year	Country	Department	Design	Ultrasound performer
Lin [105]	2018	USA	Emergency	Observational	EDP

*EDP* emergency department physician

### Financial costs

No publication regarding financial costs was identified.

### Legal consequences

We identified one publication concerning legal consequences following the use of non-radiologist point-of-care US (Table 11) [106]. This was a retrospective study concerning extent and quality of lawsuits. A search of the United States Westlaw database identified two lawsuits. Both lawsuits concerned the fact that the non-radiologist point-of-care US exam was not performed; in the first case, the placement of a peripherally inserted venous catheter in a child should have been checked with point-of-care US according to the accusers. In the second case, blood was found in the retroperitoneal space and it was claimed that a FAST exam should have been done. In both cases the defendants (i.e. the physicians) were acquitted.

**Table 11:** Possible harms

Author	Year	Country	Department	Design	Harm
Nguyen [106]	2016	USA	Neonatology, paediatrics	Retro	Two lawsuits were identified, both concerning failure to perform a point-of-care US exam. Both were won by defendants (physicians)

*Retro* retrospective

## Discussion

We conducted this scoping review to gain an overview of current uses of abdominal non-radiologist point-of-care US in children to (1) make radiologists and non-radiologists more aware of its status and (2) prompt both categories of US performers to collaborate with each other. This scoping review demonstrates that non-radiologist point-of-care US is increasingly used and studied in paediatric care for a variety of indications. It also shows that non-radiologist point-of-care US in certain indications can have a positive impact on patient care and outcome, e.g., by reducing number of CTs needed or reducing length of hospital stay. This supports the further development of non-radiologist point-of-care US, and it underlines the need for consensus on who can do which examinations.

This scoping review also assessed the quality of examinations and training of non-radiologists performing abdominal point-of-care US in children. Regarding the quality, in some settings non-radiologists performed equal to radiologists [8, 23, 29, 30], but this was certainly not always the case [20, 31]. Moreover, clinically important missed diagnoses have been reported [39], underlining the need for proper training of non-radiologists. This scoping review makes clear that no standardised training guidelines are available, which is a key issue for the further development of non-radiologist point-of-care US.

Based on the included studies, effective training could start with a short introduction lecture, followed by an online training program (e.g., Kwan et al. [101]), which can be followed at home, and such training could conclude with a non-radiologist point-of-care US rotation in the emergency department, radiology department or both. In the included studies, a basic training of just 1–2 h was found to be sufficient for physicians performing dedicated single-organ point-of-care US exams. We, however, believe that in order to gain more generalizable skills and to ensure a high quality of all operators, a more thorough approach is needed, with paediatric radiologic input. An example of how collaboration between non-radiologists and radiologists could help to maintain quality of the non-radiologist point-of-care US exams is implementing a review process, as Conlon et al. [97] described, where radiologists and non-radiologists come together on regular basis to discuss cases.

There are some important issues to take into consideration before further implementing non-radiologist point-of-care US into daily care. First, very few studies have properly looked at missed diagnoses or incorrect diagnoses. There is a risk of non-radiologist point-of-care US leading to a delayed diagnosis and subsequently to the patient’s wellbeing being at risk. The fact that these cases have not led to published lawsuits is not evidence that this is not a problem. Second, no studies exist on the financial costs of readily available point-of-care US, which could lead to an increase in health care costs; hence a proper cost–benefit analysis is warranted. Also, little attention has been paid to the patient’s perspectives thus far. In addition, few studies compared the performance of the non-radiologists to that of radiologists. Comparing a non-radiologist to a radiologist after completing a proper training program would give more insight into the quality of US examinations. Last, from the included studies we cannot conclude what the impact of is on the clinical daily practice because the studies describe research circumstances. More research on this topic is needed before implementing changes to point-of-care US usage.

The strengths of this scoping review are our thorough search strategy with help of a clinical librarian and the cooperation of both radiologists and non-radiologists. Our scoping review has some limitations as well. First, we limited our scoping review to abdominal US. This was done to keep a clear focus; however, we suspect that a similar result can be found in other fields where non-radiologist point-of-care US is being used, such as in chest or musculoskeletal US. Second, we limited our scoping review to in-hospital use of non-radiologist point-of-care US in developed countries. Our findings might have been different in low-resource countries, where access to radiology departments can be limited. In such a setting non-radiologist point-of-care US might well be the only imaging modality available. In addition, we did not perform a quality assessment of included studies because we aimed to provide a general overview and not to answer a very specific research question through a systematic review. Also, we excluded articles including both children and adults if the data could not be separated. This might have led to a loss of relevant information.

## Conclusion

This scoping review supports the further development of non-radiologist point-of-care US and underlines the need for consensus among US performers on who can do which examination after which level of training. More research on training non-radiologists and on cost–benefit of non-radiologist point-of-care US is needed.

## Supplementary Information

ESM 1 (XLSX 12 kb)
